# Repeatability and reproducibility of various 4D Flow MRI postprocessing software programs in a multi-software and multi-vendor cross-over comparison study

**DOI:** 10.1186/s12968-023-00921-4

**Published:** 2023-03-28

**Authors:** Thekla H. Oechtering, André Nowak, Malte M. Sieren, Andreas M. Stroth, Nicolas Kirschke, Franz Wegner, Maren Balks, Inke R. König, Ning Jin, Joachim Graessner, Hendrik Kooijman-Kurfuerst, Anja Hennemuth, Jörg Barkhausen, Alex Frydrychowicz

**Affiliations:** 1 Department of Radiology and Nuclear Medicine, Universität zu Lübeck, Ratzeburger Allee 160, 23538 Lübeck, Germany; 2 Center of Brain, Behavior and Metabolism (CBBM), Universität zu Lübeck, Lübeck, Germany; 3 Department of Radiology, University of Wisconsin-Madison, Madison, WI USA; 4 Institute of Medical Biometry and Statistics, Universität zu Lübeck, Lübeck, Germany; 5 Cardiovascular MR R&D, Siemens Medical Solutions USA, Inc, Cleveland, OH USA; 6 Siemens Healthcare GmbH, Lindenplatz 2, 20099 Hamburg, Germany; 7 Philips Healthcare GmbH, Röntgenstrasse 22, 22335 Hamburg, Germany; 8 Fraunhofer MEVIS, Am Fallturm 1, 28359 Bremen, Germany; 9 Institute for Imaging Science and Computational Modelling in Cardiovascular Medicine, Charité – Universitätsmedizin Berlin, Amrumer Str. 32, 13353 Berlin, Germany

**Keywords:** 4D Flow CMR, Phase-contrast magnetic resonance imaging, Flow quantification, Aorta, Blood flow velocity, Wall shear stress, Inter-scanner comparison, Inter-software comparison, Inter-rater comparison, Intra-rater comparison

## Abstract

**Background:**

Different software programs are available for the evaluation of 4D Flow cardiovascular magnetic resonance (CMR). A good agreement of the results between programs is a prerequisite for the acceptance of the method. Therefore, the goal was to compare quantitative results from a cross-over comparison in individuals examined on two scanners of different vendors analyzed with four postprocessing software packages.

**Methods:**

Eight healthy subjects (27 ± 3 years, 3 women) were each examined on two 3T CMR systems (Ingenia, Philips Healthcare; MAGNETOM Skyra, Siemens Healthineers) with a standardized 4D Flow CMR sequence. Six manually placed aortic contours were evaluated with Caas (Pie Medical Imaging, SW-A), cvi42 (Circle Cardiovascular Imaging, SW-B), GTFlow (GyroTools, SW-C), and MevisFlow (Fraunhofer Institute MEVIS, SW-D) to analyze seven clinically used parameters including stroke volume, peak flow, peak velocity, and area as well as typically scientifically used wall shear stress values. Statistical analysis of inter- and intrareader variability, inter-software and inter-scanner comparison included calculation of absolute and relative error (E_R_), intraclass correlation coefficient (ICC), Bland–Altman analysis, and equivalence testing based on the assumption that inter-software differences needed to be within 80% of the range of intrareader differences.

**Results:**

SW-A and SW-C were the only software programs showing agreement for stroke volume (ICC = 0.96; E_R_ = 3 ± 8%), peak flow (ICC: 0.97; E_R_ = −1 ± 7%), and area (ICC = 0.81; E_R_ = 2 ± 22%). Results from SW-A/D and SW-C/D were equivalent only for area and peak flow. Other software pairs did not yield equivalent results for routinely used clinical parameters. Especially peak maximum velocity yielded poor agreement (ICC ≤ 0.4) between all software packages except SW-A/D that showed good agreement (ICC = 0.80). Inter- and intrareader consistency for clinically used parameters was best for SW-A and SW-D (ICC = 0.56–97) and worst for SW-B (ICC = -0.01–0.71). Of note, inter-scanner differences per individual tended to be smaller than inter-software differences.

**Conclusions:**

Of all tested software programs, only SW-A and SW-C can be used equivalently for determination of stroke volume, peak flow, and vessel area. Irrespective of the applied software and scanner, high intra- and interreader variability for all parameters have to be taken into account before introducing 4D Flow CMR in clinical routine. Especially in multicenter clinical trials a single image evaluation software should be applied.

## Background

4D Flow cardiovascular magnetic resonance (CMR) offers the unique opportunity to gather time-resolved, 3-dimensional and 3-directional flow information of the heart and blood vessels non-invasively and without contrast agent. Using 4D Flow CMR, routinely used basic flow parameters such as stroke volume, velocities, and flow volumes can be evaluated simultaneously with matching geometric information and advanced derived parameters such as wall shear stress, pressure gradients, and turbulent kinetic energy [1]. Furthermore, 4D Flow CMR allows retrospective analysis of a 3D volume with complex vascular anatomies and flow patterns without the necessity of repeated scans and planning. This makes it an ideal technique for the evaluation of patients with complex anatomy such as congenital heart disease [2].

Reproducibility of results between both different scanners and post-processing software remains to be addressed [1, 3]. Unconfirmed reproducibility limits the possibility to perform meta-analyses and imposes bias to any multicenter trials in order to determine the clinical relevance of 4D Flow CMR and its vast number of available parameters. And while most studies nowadays comply to the scan recommendations of the consensus paper by Dyverfeldt et al. [1], there is a plethora of measured and deducted parameters extracted by a considerable number of different software applications, some commercially available, some home-built lacking thorough comparison or standardization. However, software programs must provide repeatable and reproducible results to be used interchangeably. While the variability of measurements from different sequences and vendors has been recognized [4–8], there is a relevant lack of comparative studies analyzing interchangeability of 4D Flow CMR postprocessing software.

Hence, the goal of this study was to perform a cross-over comparative study of 4D Flow CMR software analyzing data of healthy individuals’ thoracic aorta scanned on two 3 T MRI scanners of different vendors. As primary endpoint, repeatability and reproducibility of quantitative 4D Flow CMR results from four different postprocessing software programs as well as reproducibility of results between different software programs were to be evaluated. As secondary endpoint, reproducibility between MRI systems by different vendors was analyzed.

## Methods

### CMR scan

Thoracic aortic 4D Flow CMR was conducted in eight healthy subjects (27 ± 3 years, full demographics in Table 1) scanned on two 3 T CMR scanners (MRI1 = Ingenia, Philips Healthcare, Best, The Netherlands; MRI2 = MAGNETOM Skyra, Siemens Healthcare, Erlangen, Germany) using scan parameters per guideline recommendations [1]. On MRI2, a prototype 4D Flow CMR sequence was used. Acquisition settings for both sequences were carefully adapted to match each other closely (Table 2). The adaption of these parameters resulted in a nominal echo time (TE)/repetition time (TR) of 1.7/3.0 ms for MRI1 and 2.2/4.8 ms for MRI2. To exclude circadian effects on hemodynamics, both scans were acquired at the same time of day. To exclude effects of digestion, all participants fasted 2 h prior to CMR scans.

**Table 1:** Demographics

Variables	Healthy subjects (n = 8)
Age (years)	27 ± 3
Height (cm)	176 ± 6
Weight (kg)	80 ± 15
Body mass index (m^2^)	25.5 ± 3.1
Gender ratio (male: female)	5:3
Systolic blood pressure (mmHg)	131 ± 16
Diastolic blood pressure (mmHg)	80 ± 8
Heart rate (bpm)	66 ± 8

**Table 2:** Typical scan parameters

Parameter	Unit	MRI1	MRI2
Field-of-view	mm^3^	290 × 290 × 56	312 × 384 × 50
Acquired spatial resolution	mm^3^	2.5 × 2.5 × 2.5	2.5 × 2.0 × 2.5
Reconstructed spatial resolution	mm^3^	2.0 × 2.0 × 2.0	2.0 × 2.0 × 2.0
Number of reconstructed heart phases		24	24
ECG synchronization		Retrospective	Retrospective
Respiratory motion compensation		Gating window 8 mm	Gating window 8 mm
Flip Angle	°	8	7
Parallel Imaging		SENSE acceleration factor: 2.2	GRAPPA acceleration factor: 3.0
Velocity-encoding factor	cm/s	200	200

ECG, electrocardiogram

### Processing of CMR data

**Fig. 1:**
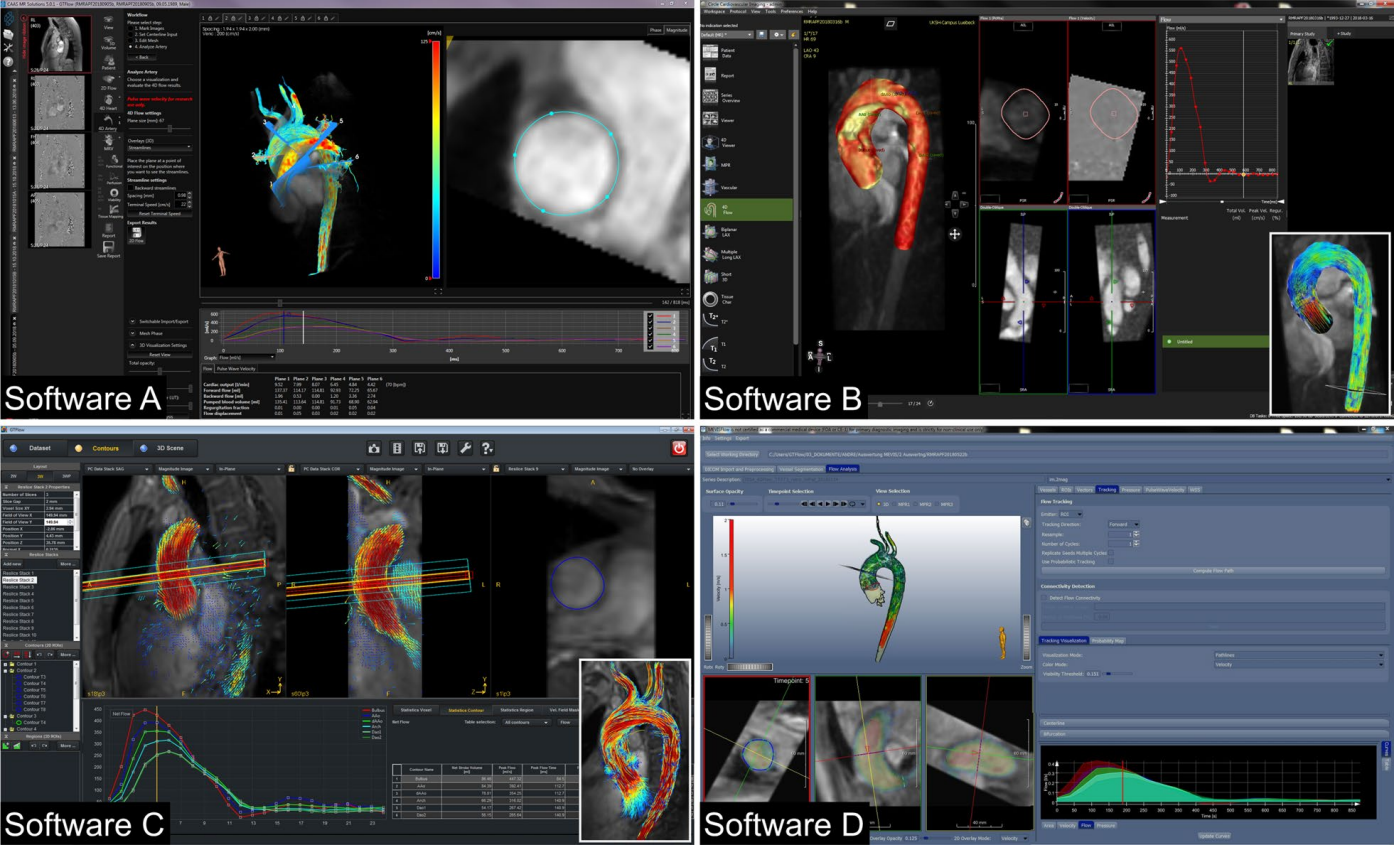
Screenshots of software A (**SW-A**): Caas (v5.01, Pie Medical, Maastricht, Netherlands), **SW-B**: cvi42 (v5.9.4, Circle Cardiovascular Imaging, Alberta, Calgary, Canada), **SW-C**: GTFlow (v3.1.13, Gyrotools, Zurich, Switzerland), and **SW-D**: MEVISFlow (v10.3, MEVIS Fraunhofer, Germany). Note that a white framed 3D visualization of the thoracic aorta is added to screenshots of software **B** and **C** in the right lower corner, since this would be displayed on a second screen. Note how the contours in **SW-A**, **SW-C**, and **SW-D** delineate the vessel wall of the ascending aorta. In contrast, the contour in **SW-B** deviates from the vessel wall as it was not possible to adjust the contour freely

The resulting 16 datasets were evaluated with four different software packages that were available at our hospital: Three programs were commercially available [SW-A = Caas (v5.01, Pie Medical, Maastricht, the Netherlands), SW-B = cvi42 (v5.9.4, Circle Cardiovascular Imaging, Calgary, Alberta, Canada), and SW-C = GTFlow (v3.1.13, GyroTools, Zurich, Switzerland), alphabetical order]. One additional program was only available through a research collaboration [SW-D = MEVISFlow (v10.3, MEVIS Fraunhofer, Germany)]. Programs were installed on the same computer (Intel® Xeon® CPU E5-1620 v3 @ 3.50 GHz processor, 16 GB RAM, NVIDIA Quadro K4200 graphic card). Sample screenshots of the graphical user interface of the individual software programs can be seen in Fig. 1.

All datasets were independently evaluated with each software by two readers who were blinded to the other software programs’ and the other reader’s results. To account for real-life scenario and potential errors, each reader underwent the entire data evaluation process for each software including data filtering, analysis plane positioning and segmentation. Both readers did not have prior experience with 4D flow CMR analysis to exclude bias by different levels of familiarity with the programs. They were trained by experienced readers and software representatives for the purpose of this study. For intrareader comparison, all datasets were re-evaluated by one reader after three to six months to avoid recognition effects. Every postprocessing workflow started with loading the DICOM data into the respective software. In each program, phase background offsets caused by eddy currents were corrected. SW-B, SW-C, and SW-D allowed manual thresholding for the selection of static tissue. Differences in background phase offset correction between the programs included different grades of polynomial fitting (SW-A and SW-B: linear; SW-C: quadratic; SW-D: cubic).

**Fig. 2:**
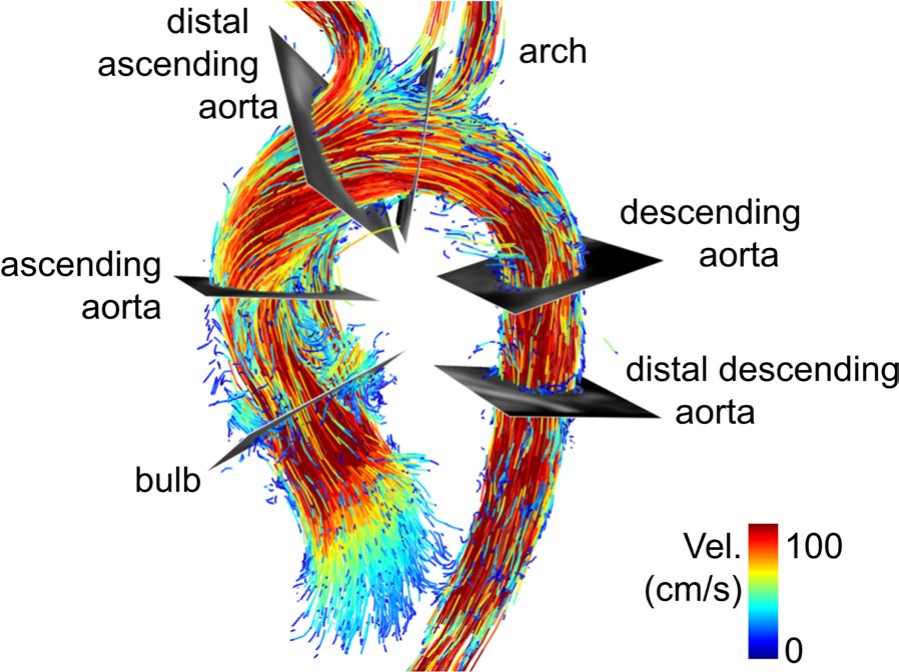
Definition of cut plane location in the thoracic aorta. Cut plane positions were defined at the aortic bulb, in the ascending aorta at the level of the pulmonary bifurcation, in the distal ascending aorta proximal to the brachiocephalic trunk, in the aortic arch between left carotid and subclavian artery, in the descending aorta at the level of the pulmonary bifurcation, and in the distal descending aorta at the level of the aortic bulb

In SW-A, SW-B, and SW-D, a 3D vessel contour of the thoracic aorta was delineated semi-manually, and voxels outside this region of interest were ignored during the analysis. In SW-B, manual changes of the contour delineation were inevitably corrected automatically. There was no way to turn off the auto-correction. This made it impossible for users to delineate the vessel exactly as they intended (Fig. 1). In SW-C, a noise masking was applied. Six contours were carefully placed manually at six predefined sites of the thoracic aorta using B-splines in multiplanar reformatted planes (Fig. 2). Contours were manually adjusted for each time frame. Typically used clinical parameters were evaluated: forward, backward, and net stroke volumes (fwSV, bwSV, netSV, respectively [ml]), peak flow [ml/s], peak maximum velocity of a contour, i.e. the highest velocity that was measured at one voxel in a contour (V_max_ [cm/s]), peak velocity averaged over a contour (V_avg_ [cm/s]), and area at time of peak flow (Area [mm^2^]). Moreover, peak values of wall shear stress (WSS [mPa]) were evaluated averaged over a contour (WSS_con_) as well as for the maximum segmental WSS of the contour that was subdivided into 8 segments (WSS_seg_). SW-A calculated WSS as described by Perinajova et al. [9]. SW-B and SW-C calculated WSS according to Stalder et al. [10]. Zimmermann et al. [6] described the approach to calculating WSS as implemented by SW-D. In SW-A, SW-C and SW-D, the viscosity of blood assumed for the WSS calculations could be manually adapted and was set to 3.2 mPa*s to match the fixed value of SW-B. SW-B offered no segmental maximum WSS values, but only WSS values averaged over the whole contour.

Different software issued different names for the evaluated parameters (Table 3). Duration of evaluation was noted for the second evaluation with every software, excluding the time needed for the import of digital imaging and communications in medicine (DICOM) data.

**Table 3:** Different software issue different names for parameters

Nomenclature of this publication	SW-A	SW-B	SW-C	SW-D
Stroke volume (ml)	Pumped blood volume (ml)	Total Volume (ml)	Stroke Volume (ml)	Flow (l)
Forward stroke volume (ml)	Forward flow (ml)	Total Forward Volume (ml)	Net Forward Flow Volume (ml)	Forward (l)
Backward stroke volume (ml)	Backward flow (ml)	Total Backward Volume (ml)	Net Backward Flow Volume (ml)	Backward (l)
Peak flow (ml/s)	Flow Graph (ml/s)	Maximum Flow (ml/s)	peak value of time resolved “Net Flow” (ml/s)	peak value of time resolved “Flow” (l/s)
Peak maximum velocity (cm/s)	Max Velocity Graph (cm/s)	peak value of time resolved “Max Mag” (cm/s)	peak value of time resolved “Velocity Max” (cm/s)	peak value of time resolved “Velocity Max” (m/s)
Peak average velocity (cm/s)	Mean Velocity Graph (cm/s)	Maximum Mean Velocity (cm/s)	peak value of time resolved „Velocity Avg “ (cm/s)	peak value of time resolved “Velocity Mean” (m/s)
Area (mm^2^)	Contour Area Graph (mm^2^)	Area (mm^2^)	Area (mm^2^)	Area (mm^2^)
Wall shear stress (WSS)				
WSS (mPa)	WSS^1,2^ (mPa)	WSS^1,2^ (Pa)	WSS Magnitude^1^ (N/m^2^)	WSS No projection^1,2,3^ (Pa)
Temporal resolution	Yes	Yes	Yes	Yes
Spatial resolution	1 contour with n (90) Vectors	1 contour, no segments	1 contour with n (8) segments	1 contour with n (8) segments
Peak WSS per segment	Calculated: 11 vectors got grouped together. Peak of those mean values	–	Peak value of time resolved, “Segment 1 WSS Mag.” to “Segment 8 WSS Mag.”	Peak value of segmentally resolved “Max WSS(mean) No Projection”
Peak WSS per contour	Calculated: Mean value of all WSS vectors	“Max Wall Shear Stress”	Peak value of time resolved “Avg WSS Mag”	Calculated: Peak value of the calculated mean WSS No Projection value of segments 1–8 calculated separately for each time point

^1^ Axial WSS also available; ^2^circumferential WSS also available; ^3^ oscillatory shear index also available

### Statistics

Repeatability and interreader reproducibility of 4D Flow CMR results were evaluated by intra- and interreader comparison, respectively. Reproducibility of results by different software programs was evaluated by inter-software comparison. To better place these results into context, reproducibility between scanners was evaluated by inter-scanner comparison determined with the software that showed best repeatability and reproducibility. As statistical measures for repeatability and reproducibility, the agreement between measurement pairs (e.g., between reader, software, scanner) was tested using Bland–Altman analysis and intraclass correlation coefficient (ICC) [11–13].

For all parameters, average absolute and relative differences, and standard deviation (SD) between two comparative pairs (methods), i.e., readers, software, or scanners, were calculated. The relative difference Δ_R(X–Y)_ for each parameter measured by methods X and Y was calculated as follows:

1 $${\Delta }_{R(X-Y)}=\frac{X-Y}{(X+Y)/2}$$

where the difference between the results of methods X and Y is divided by the mean of methods X and Y. Values are presented as mean ± SD.

Bland–Altman analyses were performed to determine the average bias and the 95% limits of agreement between each software combination for each parameter. Limits of agreement (LOA) were calculated as bias ± 1.96 * SD. In the same fashion, inter-scanner and intra- and interreader variability were statistically evaluated.

ICC estimates and their 95% confidence intervals (CI) for intrareader comparison were calculated based on a single rating, absolute-agreement, 2-way mixed-effects model. For interreader, inter-software, and inter-scanner comparisons, ICC estimates and their 95% CI were calculated based on a single rating, absolute-agreement, 2-way random-effects model [14]. ICC values less than 0.5, between 0.5 and 0.75, between 0.75 and 0.9, and greater than 0.9 are indicative of poor, moderate, good, and excellent agreement, respectively [14].

Using the described ICC estimates and results from the Bland–Altman analysis, we aimed at determining equivalence between software programs. For this, we adapted the equivalence test proposed by Zange et al. [15] to include the degree of intrareader variability in the assessment of reproducibility between software packages. Specifically, we based our assessment of differences between software on the intrareader variability: inter-software variability cannot be lower than intrareader variability of one single software. We considered software programs to be equivalent if X% of comparisons between two software programs were within the limits of agreement obtained from the intrareader variability. We determined the threshold X based on results from the interreader comparison: We determined the percentage of measurement comparisons between two readers that were within the 95% limits of agreement from the intrareader comparison, focusing only on the clinically used parameters area, stroke volume, flow, and velocity. Therefore, the narrowest limits of agreement obtained from the intrareader variability derived from Bland–Altman analysis were chosen and defined as equivalence limits. Equivalence was concluded for a parameter if X% of the absolute bias results between two software packages were completely within the limits of equivalence and ICC analysis displayed at least moderate agreement. SPSS (version 26.0 Statistical Package for the Social Sciences, International Business Machines, Inc., Armonk, New York, USA) was used for statistical analysis.

## Results

**Fig. 3:**
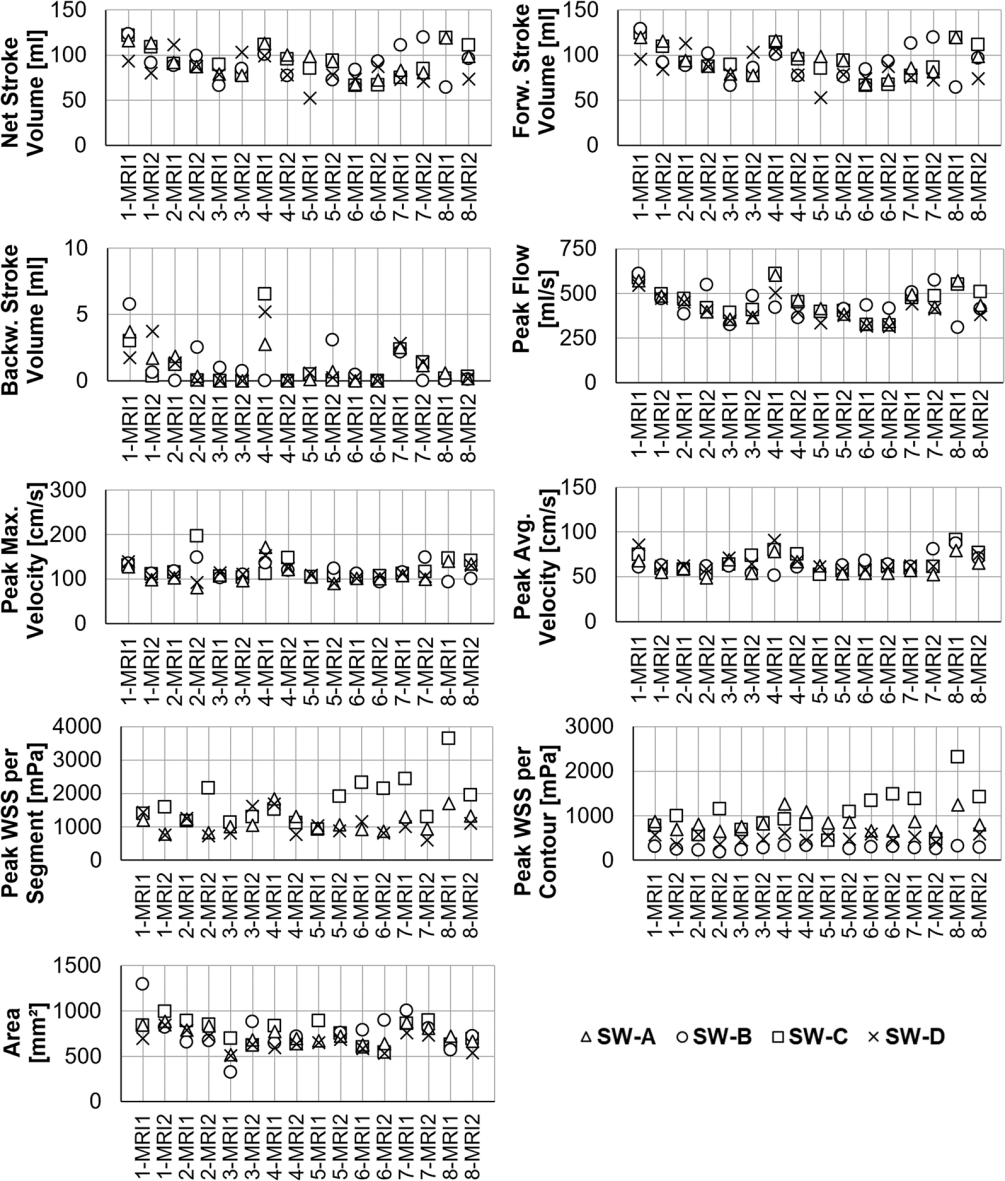
Results of the ascending aorta contour per scanner and software. Measurement results for every subject (1–8) at both scanners (MRI1, MRI2). The graphs allow the appreciation of variability between software programs and between the acquisitions at both CMR systems for each volunteer separately. Analyses included net, forward (forw.), and backward (backw.) stroke volume, peak flow, peak maximum and average (avg.) velocity, peak wall shear stress (WSS) per segment and contour, as well as area. Note the high variability of results between software solutions and scan-rescan for WSS

All eight subjects were successfully scanned at both scanners. Acquisition time was 12.4 ± 2.5 min for MRI1 and 15.7 ± 5.5 min for MRI2. Respiratory navigator acceptance rate was 55 ± 17%. SW-B and SW-D failed to open one of eight scans from MRI1, although not the same dataset concerned. There were no problems evaluating these datasets with the other software programs. Standard customer support of the respective software programs was contacted but could not resolve the issue. All other datasets could be analyzed. There was no aliasing and therefore no need for aliasing correction. Figure 3 depicts data of the ascending aortic contour for all volunteers, scanners, and software.

### Intrareader variability as measure of repeatability

Intrareader variability between software programs varied considerably. As detailed in Table 4, the intrareader analysis of SW-A revealed best repeatability with good to excellent agreement for all parameters and lowest standard deviation of relative errors between 7 and 15%. Bland–Altman analysis revealed smallest bias and narrowest limits of agreement for intrareader variability of most parameters using SW-A (e.g., LOA for stroke volume: − 12 to 12 ml, peak maximum velocity: − 23 to 21 cm/s, area: − 175 to 205 mm^2^). SW-C and SW-D showed moderate to excellent agreement except for SW-C concerning peak maximum velocity and WSS values, where ICC revealed poor agreement.

**Table 4:** Intrareader variability for all contours combined

	SW-A	SW-B	SW-C	SW-D
*Stroke volume*				
Absolute error [ml]	**0 ± 6**	− 1 ± 18	2 ± 6	0 ± 12
Relative error [%]	1 ± 7	− 2 ± 26	3 ± 8	0 ± 16
ICC	0.967***	0.648*	0.964***	0.790**
*Peak flow*				
Absolute error [ml/s]	**4 ± 27**	− 3 ± 89	26 ± 92	5 ± 41
Relative error [%]	1 ± 7	− 1 ± 27	8 ± 27	1 ± 11
ICC	0.969***	0.644*	0.663*	0.917***
*Peak maximum velocity*				
Absolute error [cm/s]	** − 1 ± 11**	− 1 ± 28	5 ± 26	− 2 ± − 20
Relative error [%]	− 1 ± 8	− 1 ± 21	4 ± 19	− 1 ± 13
ICC	0.883**	0.219	0.348	0.746*
*Peak average velocity*				
Absolute error [cm/s]	− 1 ± 10	− 2 ± 17	9 ± 10	**3 ± 7**
Relative error [%]	− 1 ± 14	− 2 ± 21	12 ± 13	4 ± 8
ICC	0.809**	0.394	0.541*	0.839**
*Area*				
Absolute error [mm^2]^	15 ± 97	13 ± 190	− 80 ± 143	** − 21 ± 87**
Relative error [%]	3 ± 15	2 ± 29	− 12 ± 19	− 4 ± 15
ICC	0.919***	0.705*	0.788**	0.915***
*Peak WSS per segment*				
Absolute error [mPa]	**8 ± 133**	–	737 ± 847	− 137 ± 614
Relative error [%]	1 ± 9	–	44 ± 50	− 5 ± 29
ICC	0.921***	–	0.068	0.677*
*Peak WSS per contour*				
Absolute error [mPa]	14 ± 121	** − 32 ± 54**	567 ± 563	− 12 ± 156
Relative error [%]	2 ± 11	− 7 ± 12	60 ± 56	− 1 ± 22
ICC	0.921***	0.944***	0.068	0.706*

Absolute and relative error are given as mean ± standard deviation. Values of bias and standard deviation yielding to smallest limits of agreement within one row are marked as bold indicating that they were used to calculate equivalence limits. Asterisks indicate ***excellent, **good, and *moderate intraclass correlation (ICC). No asterisk indicates poor agreement. WSS, wall shear stress

SW-B yielded the worst repeatability with poor to moderate agreement for most parameters, with standard deviations for stroke volume, peak flow, and peak velocities varying between 21 and 27%. This is underlined by broadest limits of agreement that were found with SW-B for stroke volume (− 36 to 34 ml), peak velocities (e.g., peak maximum velocity: − 56 to 54 cm/s), and area (− 359 to 385 mm^2^). Relative error of bwSV was high in all cases due to small absolute values, while absolute error of bwSV was low with every software.

### Interreader variability as measure of reproducibility

As expected, interreader agreement was lower than intrareader agreement with few exceptions. Lowest interreader variability was found with SW-A with moderate to excellent agreement for all parameters yielding to standard deviations of differences between readers between 10 and 22% (Table 5). Smallest limits of agreement between two readers were detected with SW-A for stroke volumes (SV: − 19 to 17 ml), peak flow (F: − 81 to 67 ml/s), and peak velocities (V_max_: − 32 to 30 cm/s), closely followed by SW-D, which displayed narrowest limits of agreement for area (A: − 65 to 229 mm^2^).

**Table 5:** Interreader variability for all contours combined

	SW-A	SW-B	SW-C	SW-D
*Stroke volume*				
Absolute error [ml]	− 1 ± 9	0 ± 20	7 ± 12	− 1 ± 12
Relative error [%]	− 1 ± 11	0 ± 28	9 ± 15	− 2 ± 16
ICC	0.920***	0.569*	0.823**	0.809**
*Peak flow*				
Absolute error [ml/s]	− 7 ± 38	− 5 ± 100	37 ± 45	1 ± 45
Relative error [%]	− 2 ± 10	2 ± 32	11 ± 12	1 ± 16
ICC	0.937***	0.565*	0.862**	0.907***
*Peak maximum velocity*				
Absolute error [cm/s]	− 1 ± 16	14 ± 27	3 ± 28	− 2 ± 19
Relative error [%]	− 1 ± 13	12 ± 23	2 ± 19	− 1 ± 13
ICC	0.705*	0.274	0.359	0.730*
*Peak average velocity*				
Absolute error [cm/s]	2 ± 14	1 ± 17	− 6 ± 10	2 ± 10
Relative error [%]	2 ± 22	1 ± 22	− 7 ± 13	3 ± 12
ICC	0.556*	0.304	0.699*	0.754**
*Area*				
Absolute error [mm^2]^	− 18 ± 156	15 ± 192	92 ± 146	82 ± 75
Relative error [%]	− 6 ± 22	0 ± 30	17 ± 22	− 6 ± 19
ICC	0.757**	0.605*	0.671*	0.861**
*Peak WSS per segment*				
Absolute error [mPa]	4 ± 190	–	599 ± 718	591 ± 755
Relative error [%]	0 ± 14	–	32 ± 42	18 ± 37
ICC	0.827**	–	0.227	0.480
*Peak WSS per contour*				
Absolute error [mPa]	31 ± 160	50 ± 78	357 ± 513	118 ± 135
Relative error [%]	3 ± 15	10 ± 18	29 ± 52	− 3 ± 26
ICC	0.857**	0.832**	0.221	0.573*

Absolute and relative error are given as mean ± standard deviation. Asterisks indicate ***excellent, **good, and *moderate intraclass correlation (ICC). No asterisk indicates poor agreement

Highest interreader variability was found with SW-B presenting poor to moderate agreement concerning clinical parameters. Broadest limits of agreement were found with SW-B for stroke volumes (SV: − 39 to 39 ml), peak flow (F: − 201 to 191 ml/s), peak velocities (V_max_: − 39 to 67 cm/s) and area (A: − 361 to 391 mm^2^). Highest bias was found with SW-C for stroke volumes, peak flow and area indicating systematic error between readers (Table 5).

In general, stroke volume and peak flow presented better reproducibility (moderate to good agreement) compared to peak velocities, which mostly displayed poor to moderate agreement between readers. WSS values displayed good agreement between both readers when they were using SW-A or SW-B, while SW-C and SW-D yielded mostly poor agreement.

### Inter-software variability as measure of reproducibility

#### Stroke volumes

**Fig. 4:**
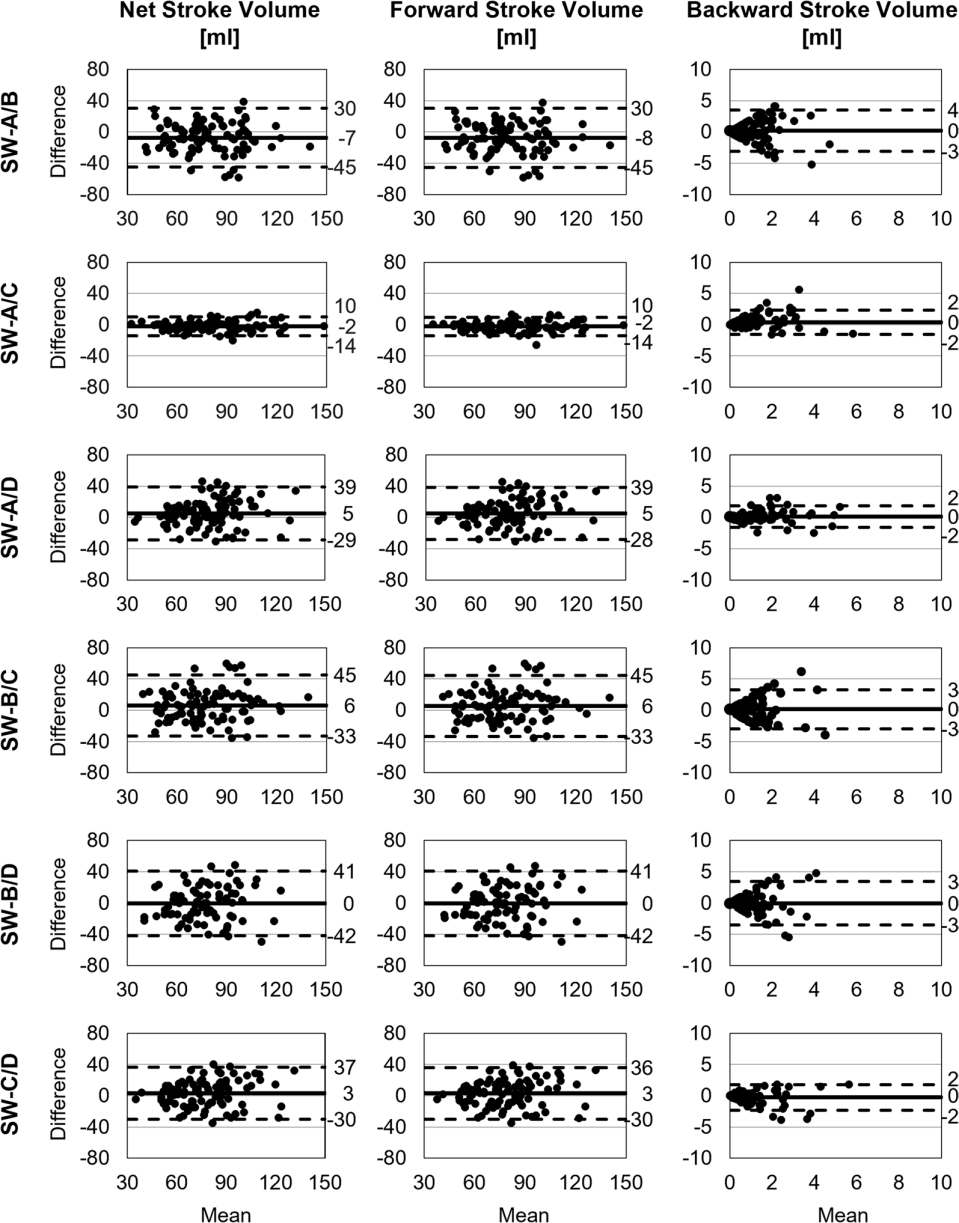
Software comparison: Bland–Altman plots of net, forward and backward stroke volume

Only SW-A/C showed an excellent agreement for netSV and fwSV with smallest bias and narrowest limits of agreement (SV: LOA = − 14 to 10 ml, E_R_ = 3 ± 8%) in the Bland–Altman analysis (Table 6, Fig. 4). In contrast, poor agreement was found with SW-B/D associated with broadest limits of agreement (SV: LOA = − 41 to 41 ml; E_R_ = − 1 ± 28%), whereas other software pairs reached moderate agreement. SW-A/D, SW-B/C and SW-C/D presented with relatively broad LOA (lower limits of agreement LLOA: − 45 to − 14 ml; upper limits of agreement ULOA: 10–33 ml). Absolute errors for bwSV were small with high relative errors due to small absolute values. Limits of agreement for bwSV varied between − 4 and 4 ml.

**Table 6:** Software comparison: absolute and relative error and correlation coefficient

	SW-A/B	SW-A/C	SW-A/D	SW-B/C	SW-B/D	SW-C/D
*Net stroke volume*						
Absolute error [ml]	7 ± 19	2 ± 6	5 ± 17	6 ± 20	0 ± 21	3 ± 17
Relative error [%]	9 ± 26	3 ± 8	6 ± 21	− 7 ± 27	− 1 ± 28	3 ± 21
ICC	0.591*	0.959***	0.634*	0.589*	0.466	0.664*
*Peak flow*						
Absolute error [ml/s]	24 ± 92	5 ± 27	17 ± 25	− 29 ± 92	4 ± 78	22 ± 25
Relative error [%]	7 ± 26	− 1 ± 7	5 ± 7	− 8 ± 26	1 ± 23	6 ± 7
ICC	0.631*	0.971***	0.972***	0.645*	0.718*	0.971***
*Peak maximum velocity*						
Absolute error [cm/s]	− 5 ± 27	− 13 ± 28	− 5 ± 14	− 10 ± 26	2 ± 28	8 ± 29
Relative error [%]	− 4 ± 21	− 10 ± 20	− 4 ± 10	− 4 ± 19	1 ± 20	6 ± 20
ICC	0.258	0.248	0.797**	0.401	0.352	0.328
*Peak average velocity*						
Absolute error [cm/s]	− 8 ± 18	− 6 ± 15	− 8 ± 10	1 ± 17	− 1 ± 17	− 3 ± 11
Relative error [%]	− 12 ± 25	− 9 ± 21	− 13 ± 15	1 ± 21	− 2 ± 22	− 4 ± 14
ICC	0.276	0.505*	0.697*	0.314	0.314	0.688*
*Area*						
Absolute error [mm^2^]	31 ± 174	21 ± 147	66 ± 84	1 ± 207	43 ± 160	48 ± 131
Relative error [%]	5 ± 26	2 ± 22	12 ± 14	− 1 ± 32	8 ± 27	10 ± 21
ICC	0.745*	0.805**	0.898**	0.614*	0.740*	0.807**
*Peak WSS per segment*						
Absolute error [mPa]	–	395 ± 760	212 ± 437	–	–	505 ± 789
Relative error [%]	–	− 17 ± 45	20 ± 31	–	–	32 ± 49
ICC		0.168	0.371			0.104
*Peak WSS per contour*						
Absolute error [mPa]	716 ± 195	64 ± 553	595 ± 244	696 ± 496	− 105 ± 126	502 ± 463
Relative error [%]	88 ± 16	14 ± 52	68 ± 22	− 77 ± 46	− 23 ± 26	50 ± 47
ICC	0.134	0.192	0.103	0.085	0.542*	0.092

Absolute and relative error are given as mean ± standard deviation. Asterisks indicate ***excellent, **good, and *moderate intraclass correlation (ICC). No asterisk indicates poor agreement

#### Peak flow

**Fig. 5:**
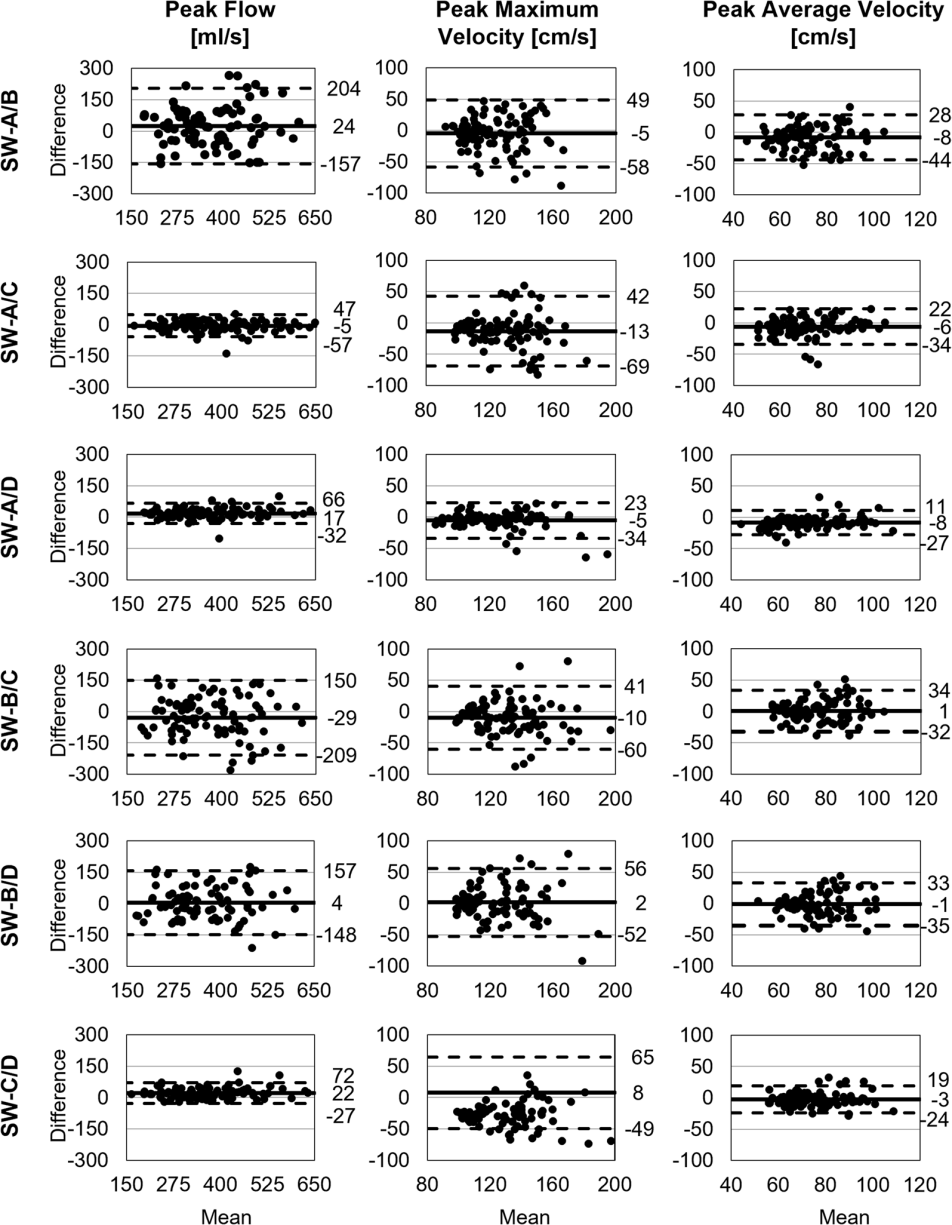
Software comparison: Bland–Altman plots of peak flow, peak maximum velocity, and peak average velocity

There was an excellent agreement for peak flow between SW-A/C, SW-C/D, and SW-A/D with relative errors in the range of − 1 to 6 ± 7% (Table 6). Bland–Altman analysis revealed narrowest limits of agreement for SW-A/D (− 32 to 66 ml/s), followed by SW-CD and SW-A/C (− 48 to 58 ml/s; Fig. 5). Compared to SW-B, all other software agreed moderately with relative differences of up to − 8 ± 26% (SW-A/B).

#### Peak velocities

There was poor agreement between all software packages for peak maximum velocity, except for SW-A/D, which agreed well and presented with narrowest limits of agreement (LOA: − 32 to 22 cm/s, E_R_: − 4 ± 10%; Table 6, Fig. 5). All other software pairs displayed high standard deviation in the range of 19–21%.

Moderate agreement was found for peak average velocity among SW-A, SW-C, and SW-D. In contrast, there was poor agreement between SW-B and the other software programs. Limits of agreement for peak average velocity were narrower than for peak maximum velocity (e.g., for SW-A/C; V_avg_: − 23 to 35 cm/s, V_max_: − 42 to 68 cm/s).

#### Area

**Fig. 6:**
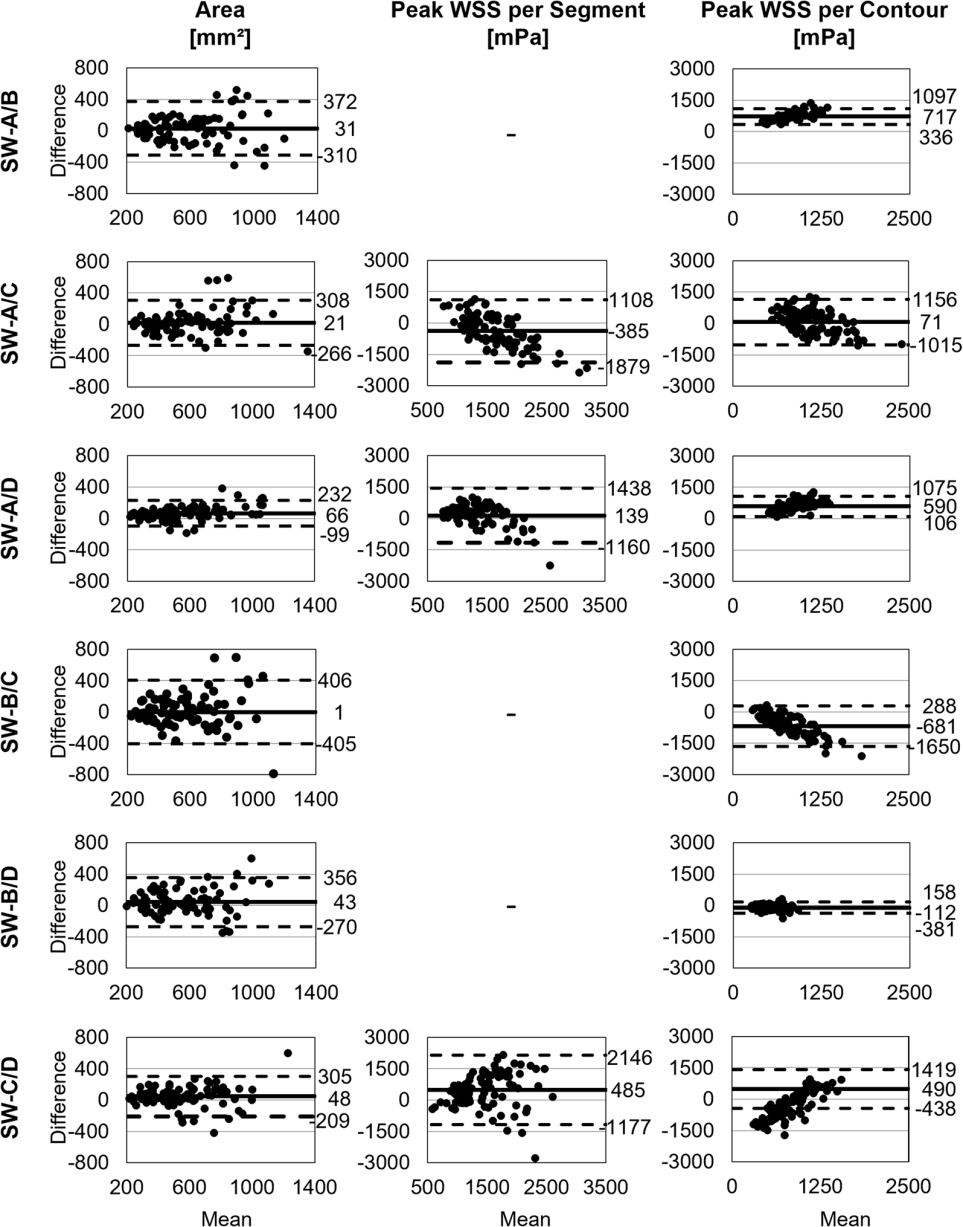
Software comparison: Bland–Altman plots of area, peak wall shear stress (WSS) per segment and peak WSS per contour

There was good agreement for maximum area among SW-A, SW-C, and SW-D (Table 6). SW-B showed moderate agreement with the other software programs. The smallest absolute bias but broadest limits of agreement for the contour area was between SW-B and SW-C (E_A_ = − 1 ± 207 mm^2^; LOA: − 407 to 405 mm^2^; Fig. 6). Narrowest limits of agreement were found for SW-A/D (− 99 to 230 mm^2^), followed by SW-C/D. Both readers noted independently that SW-B inevitably automatically adapted the manually corrected contour. Due to this automated correction, it was not possible to delineate the vessel contour in exactly the way they wanted to.

#### Wall shear stress (WSS)

There was a moderate positive correlation for the peak WSS per contour between SW-B/D with high relative error (E_R_ = − 23 ± 26%; Table 6). All other software pairs showed poor agreement for both WSS parameters (ICC ≤ 0.37) with relative bias of up to 88 ± 16% (WSS_con_ with SW-A/B). Bland–Altman analysis showed smaller bias and smaller limits of agreement for peak WSS per contour compared to peak WSS per segment (Fig. 6).

#### Equivalence test

81–85% of measurements between two readers for the clinically used parameters (area, SV, flow, velocity) were within 95% limits of agreement from intrareader comparison when they used the same software. We have therefore chosen a clear-cut threshold of X = 80%. Equivalence was concluded for a parameter if values between the 10th and 90th percentile of absolute bias between two software packages, i.e., 80% of values, were completely within the limits of equivalence.

**Fig. 7:**
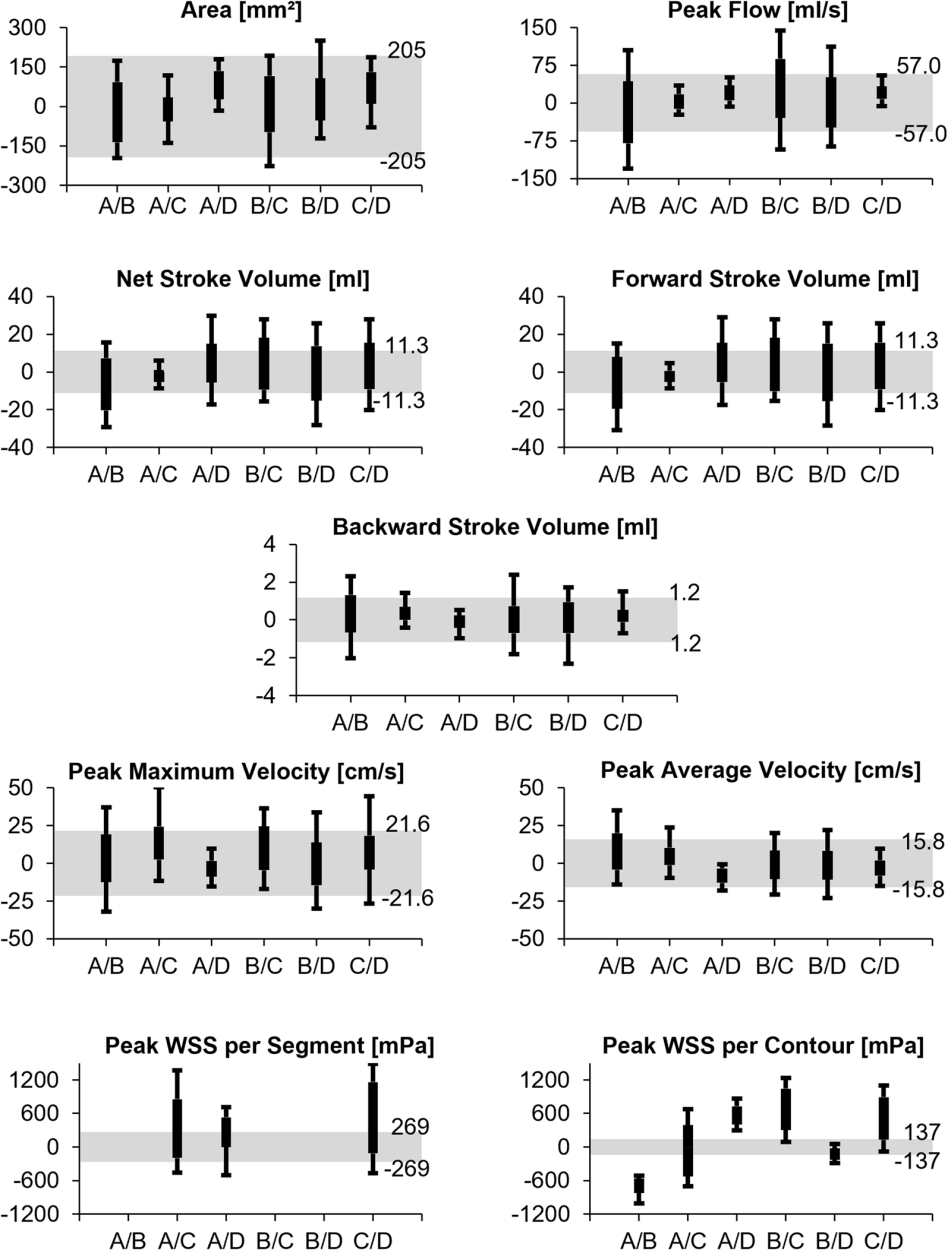
Software comparison: equivalence test. Equivalence interval, derived from smallest limits of agreement of intrareader comparison, shaded in grey. Box plots indicate 25% and 75% percentile with whiskers from 10 to 90% percentile. Whiskers, i.e. 80% of comparisons between two software, were to lie within the equivalence interval for two software packages to be considered equivalent

SW-A, SW-C and SW-D all together reached equivalence regarding area and peak flow (Fig. 7). Less than 80% of comparisons including SW-B were within the equivalence limits, thus not reaching equivalence for area and peak flow. Only SW-A/C reached equivalence for stroke volume but failed to prove equivalent for peak velocities. No software pair could prove equivalent for both, peak average, and maximum velocity. 80% of differences between SW-C and SW-D were within equivalence levels for peak average velocity, while for peak maximum velocity this was the case with SW-A/D. There was no equivalence between software for WSS.

#### Duration of evaluation

Evaluation was fastest with SW-A (22 ± 7 min) and SW-D (23 ± 6 min), followed by SW-C (36 ± 11 min). It must be noted that SW-C crashed 1.4 times per evaluation and had to be restarted, increasing overall evaluation time by an average of 12 ± 4 min. This error was fixed in the following software version which should therefore allow for an evaluation of about 24 min. Based on these results, there would be no significant differences between SW-A, SW-C, and SW-D in terms of evaluation duration (for all, p > 0.05). Processing time using SW-B was significantly prolonged due to an inevitable segmentation algorithm that repeatedly changed manually drawn contours (51 ± 10 min, for all, p ≤ 0.01).

### Inter-scanner variability as measure of reproducibility

Heart rate and blood pressure for both measurements showed only minor differences that revealed no statistical significance (MRI1: 66 ± 8 bpm, 131 ± 16/80 ± 8 mmHg; MRI2: 66 ± 13 bpm, 133 ± 16/83 ± 8 mmHg; for all, p > 0.05). Scan duration was 12 ± 3 min at MRI1 and 14 ± 1 min at MRI2 (p = 0.23). Time between both scans was 104 ± 59 days. For inter-scanner comparison, SW-A was chosen due to its comparably small intra- and interreader variability.

**Fig. 8:**
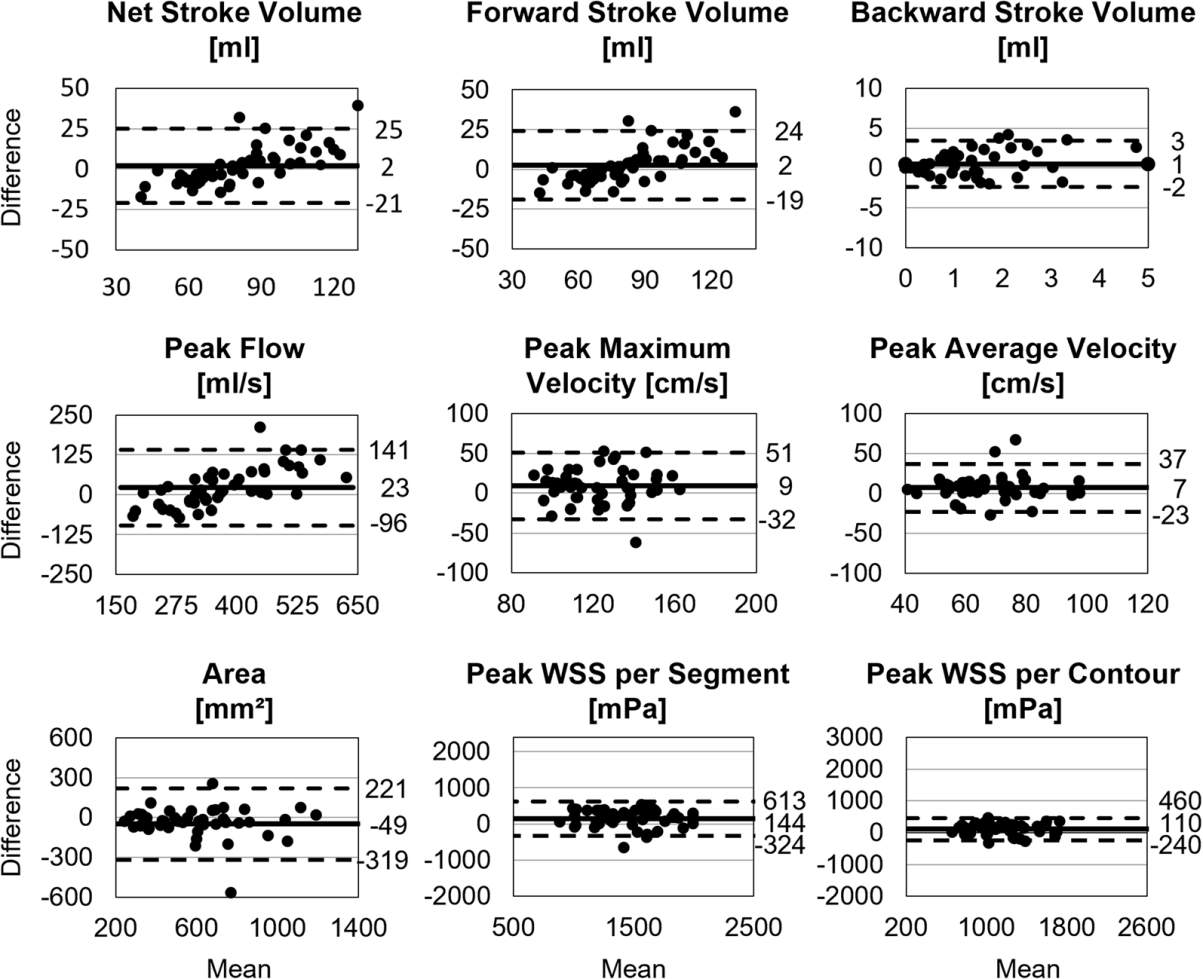
Scanner comparison: Bland–Altman plots for results by MRI1 and MRI2

There was good agreement for netSV and fwSV between MRI1 and MRI2 (Table 7) with a relative error of 0 ± 15% and limits of agreement between − 22 and 26 ml for netSV (Fig. 8). Limits of agreement of bwSV were − 3 to 1 ml. Between scans, there was a good agreement for peak flow (E_R_ = 3 ± 16%, LOA − 96 to 140 ml/s). Regarding peak velocities, there was a low agreement between both scans with relatively high relative error of 8 ± 17%. There was better agreement, lower bias and narrower limits of agreement for peak average velocity compared to peak maximum velocity (LOA; V_max_: − 32 to 50 cm/s, V_avg_: − 22 to 36 cm/s). There was good agreement for the vessel area between scans with a systematic underestimation of − 8 ± 20% on MRI1 data compared to MRI2. Between both scans, there was good and moderate agreement for peak WSS per contour and per segment, respectively, with a relative error of 10 ± 16% and 11 ± 17%, respectively.

**Table 7:** Scanner comparison: absolute and relative error and correlation coefficient, evaluated with SW-A

	Stroke volume	Forward stroke volume	Backward stroke volume
Absolute error	2 ± 12 ml	2 ± 11 ml	− 1 ± 1 ml
Relative error [%]	0 ± 15	1 ± 13	38 ± 120
ICC	0.862**	0.874**	0.337

	Peak flow	Peak maximum velocity	Peak average velocity
Absolute error	22 ± 60 ml/s	9 ± 21 cm/s	7 ± 15 cm/s
Relative error [%]	3 ± 16	8 ± 17	11 ± 22
ICC	0.832**	0.474	0.491

	Area	Peak WSS per segment	Peak WSS per contour
Absolute error	− 49 ± 137 mm^2^	144 ± 239 mPa	110 ± 179 mPa
Relative error [%]	− 8 ± 20	11 ± 17	10 ± 16
ICC	0.828**	0.659*	0.764**

Absolute error = MRI1–MRI2. Relative error = absolute error / 0.5* (MRI1 + MRI2). Absolute and relative errors are given as mean ± standard deviation. Asterisks indicate ***excellent, **good, and *moderate intraclass correlation (ICC). No asterisk indicates poor agreement

## Discussion

### Intrareader repeatability and interreader reproducibility

#### Focus on software

There were relevant differences of intra- and interreader variability between software packages with limits of agreement varying by factors between 1.9 and 4.5. SW-A presented with highest repeatability and reproducibility for most parameters, followed by SW-D and SW-C. Repeatability and reproducibility were worst with SW-B except for WSS. We hypothesize that the inevitable automated correction of vessel contours plays a major role in the explanation for the poor performance of SW-B. Ideally, semi-automated contour detection should decrease evaluation time as well as intra- and interreader variability. In this study, the opposite was the case for SW-B. Interestingly, SW-B showed good agreement for WSS in the intra- and interreader comparisons although repeatability of contour delineation was low. This was unexpected as WSS is very sensitive to contour delineation [10].

#### Focus on parameters

SV, peak flow, and area showed good to excellent repeatability and reproducibility with most software packages. In comparison, peak velocities and WSS displayed less repeatability and reproducibility. A higher variability of maximum velocity and WSS results was expected. Both parameters are highly dependent on a quantity of parameters such as noise, temporal and spatial resolution, plane positioning, as well as contour delineation [10, 16]. Moreover, maximum velocity values depend on one single voxel that presents with the highest velocity value. There is no averaging in time nor space to mitigate these interfering factors. This may explain why peak maximum velocity and WSS showed worse repeatability and reproducibility than averaged parameters.

#### Comparison to the literature

Other 4D Flow CMR studies found dramatically better interreader agreement. Typically, bias between two readers for SV varied between 0 and 3 ml with a standard deviation between 2 and 4 ml [10, 17, 18], compared to the here presented bias of − 1 to 7 ml (SD 9–20 ml). Peak flow interreader variability was published to be − 8 ± 12 ml/s [18] compared to this study’s best result with SW-A of − 7 ± 38 ml/s. Another group found an interreader bias for vessel area of 45 ± 14 mm2 [10] compared to this study’s best result of 82 ± 75 mm^2^ with SW-D.

However, in these studies [10, 17, 18] both readers used the same analysis plane and same eddy current corrections for the vessel segmentation. In contrast, we did not use the same plane for comparisons. Every reader applied background phase offset correction and noise reduction individually and used multiplanar reformatting with every single software to find the adequate planes and adjusted the contour for every single timestep. This much closer reflects “real life” and is better suited for comparison of results from different groups than inter- and intrareader comparisons undertaken on exactly the same image.

We found only one paper that explicitly described “background correction and measurements” undertaken by two readers independently to determine the interreader reproducibility of thoracic 4D Flow CMR. In contrast to our study, Chelu et al. used gadolinium-based contrast agents, General Electric Healthcare CMR scanners (General Electric Healthcare, Chicago, Illinois, USA), and another post-processing software (Arterys Inc, San Francisco, California, USA). However, they found a comparable correlation for stroke volume in patients’ ascending aorta with an unspecified intraclass coefficient of 0.975 [19], compared to an ICC of 0.920 for stroke volume of all contours in the thoracic aorta with SW-A. Another study of intracranial 4D Flow CMR using Arterys software (Arterys, Inc.) found excellent interreader agreement (ICC > 0.9) for blood flow and peak velocity [20]. Using SW-C and SW-D, we could reproduce closely matching results in the thoracic aorta for blood flow but not for peak velocity. However, the methods cannot be compared directly since Wen and colleagues [20] measured carotid and intracranial flow, which are inherently less prone to motion artifacts than thoracic or abdominal exams, and they averaged three consecutive slices.

A different study found good to excellent intra- and interreader reproducibility for regional aortic WSS_seg_ (ICC ≥ 0.78) and WSS_con_ (ICC ≥ 0.86) using SW-A [21]. We registered similar reproducibility for peak WSS_seg_ only with SW-A (ICC = 0.83) and for peak WSS of a contour only with SW-A and SW-B (ICC ≥ 0.706).

Given the high variability of measurements, plane-wise analysis seems not optimal for evaluation of complex 4D Flow CMR data since it can miss peak values. This affects particularly parameters that have a high variation within one vessel segment and are sensitive to noise, such as the peak velocity and WSS [10, 16]. One option to mitigate the effect of noise might be to use the median value of measurement on three consecutive slices [20], as it was also recommended by the Quantitative Imaging Biomarker Alliance (QIBA), [12]. On top of that, 3D analysis with voxel-by-voxel analysis and maximum intensity projection should perform better for detection and localization of peak values [22]. However, this was not available with the tested software programs.

### Reproducibility of different postprocessing software

#### Focus on software

While SW-A, SW-C, and SW-D produced equivalent results for area and peak flow, only SW-A/C proved equivalent for stroke volume. The reason for the high deviation of SW-B from results with other software packages lies probably in the automated correction of contours that did not allow an undisturbed manual delineation of the contour. In general, we suspect differences between software packages at least partly to originate from different background phase correction methods, temporal and spatial smoothing, and different interpolation algorithms of each software.

It must be noted that the equivalence test allowed a broad spectrum of errors due to the relatively high intrareader variability of all software packages. However, given the relatively high intrareader variability, it would not have made sense to choose narrower limits of equivalence.

#### Focus on parameters

Peak flow, stroke volume, and area were the parameter with the best reproducibility among the tested software packages, matching their good repeatability and interreader reproducibility. For the same reasons, i.e. noise, resolution, plane positioning and contour delineation, peak velocities and WSS were the parameters with the worst reproducibility among software programs.

#### Comparison to the literature

Compared to a recently published 2D phase contrast (PC) CMR on differences between various software programs, the bias and limits of agreement of the herein presented results were remarkably high. Typical 2D PC CMR values [bias ± SD] were reported: maximum velocity: 0–5 ± 3 cm/s for stroke volume: 0–3 ± 3 ml between three software programs [15]. However, hemodynamic measurements are dependent on the positioning of the plane [23]. This influencing factor was not considered in the 2D PC CMR study since the same plane was analyzed with different software packages. In our study, plane positioning remained an influencing factor adding to the potential sources of error. Additionally, other sources of error including elaborate postprocessing in 4D Flow CMR do not apply for 2D PC CMR. Further, noise is higher in 4D Flow CMR data because of the typically two- to three-fold smaller voxel size compared to 2D PC CMR. Therefore, differences between software programs or readers using the same DICOM data set were expected to be smaller for 2D PC CMR compared to 4D Flow CMR.

### Reproducibility of different scanners

Of note and counterintuitively, data from different scanners evaluated with a single software typically resulted in better comparability than data from a single scanner evaluated with different software programs. This was especially true for WSS: There was moderate to good agreement between both scans while—apart from SW-B/D—WSS values did poorly agree between different software packages. In general, the scanner comparison yielded similar or lower bias, but higher standard deviation compared to software comparison. Higher standard deviations were expected since both scans were done months apart and it was anticipated that physiological changes would alter the results between both scans.

However, the presented smaller interscan bias is surprising, given that previous studies showed significant differences between different scanners of the same vendor and between vendors. For 2D PC measurements, the velocity offset between measurements with the same scanner and sequence at different sites is well acknowledged [24]. Similarly, Bock et al. have shown a decreased bias for kinetic energy evaluation if scans were repeated with an interval of 14 days at the same scanner as opposed to a repeat scan using a scanner from a different vendor on the same day [4].

Scan-rescan abdominal 4D Flow CMR studies revealed neglectable bias for stroke volume (0 ± 3 ml) [25] and flow (2 ± 5 ml/min) [26], evaluating scans performed on the same day using the same scanner, while there were larger limits of agreement of scans performed on different days (flow 0 ± 11 ml/min), [26] - presumably due to physiological fluctuations. The impact of physiological variability to the inter-scanner variability in this study should therefore not be underestimated.

Van der Palen et al. [21] found moderate to good agreement for mean and maximum WSS between two consecutive scans performed on the same scanner. Similar agreement was achieved in our study for WSS values derived from two different scanners underlining good comparability of the sequences at both scanners.

### Clinical relevance

Ultimately, our results underline that only SW-A and SW-C can be used equivalently for determination of stroke volume, peak flow, and area. One can neither compare results from the other tested 4D Flow CMR analysis software programs nor can one compare the other tested parameters between software packages. Unexpectedly, results from different scanners often showed better agreement than results from different software packages. This is promising, since careful adaption of sequences at scanners of different manufactures may allow for multicenter studies with scans from different scanners in the future using the same postprocessing software.

Inter- and intrareader consistency was best with SW-A and SW-D that also permitted fastest evaluation times. WSS results should be interpreted cautiously due to low repeatability and reproducibility between software programs. Relatively high intra- and interreader variability do not allow the calculation of a conversion factor between programs at the moment. Overall high variability of peak velocities with all software packages are alarming since this parameter is frequently used in clinical routine for stenosis classification.

The authors of this paper identified the following issues that should be addressed by the software vendors to improve repeatability and reproducibility of 4D Flow CMR results:

1. Making analysis less susceptible to minor changes of contour placement and vessel wall delineation. This could be achieved by intelligent averaging algorithms over several contours and improved vessel wall detection. Further, the analysis of peak values by volume maximum intensity projection analyses might reduce the risk of underestimated peak values due to misplacement of the analysis plane. An automatic averaging of results from three consecutive planes as recommended by the Quantitative Imaging Biomarker Alliance (QIBA) [12] might reduce bias related to noise.
2. Homogenizing masking and correction methods for background phase offsets and interpolation methods for oblique cut planes. Disclosure of underlying concepts and equations for basic and advanced postprocessing steps would facilitate comparability between software.

Additional studies are needed to identify the sources of the differences in results between software solutions. For example, flow analysis in a standardized imaging plane could help to assess the impact of plane selection on results. Further optimization and standardization of software results and workflows is necessary to achieve effective comparison and expand the applicability of 4D Flow CMR findings.

### Limitations

We acknowledge that results from a small group of young and healthy subjects cannot be directly transferred to patients. Future studies should comprise patients and the analysis of variations such as diurnal or postprandial changes. Moreover, we regarded the error in different areas of the thoracic aorta as equivalent—in a larger cohort, it would be interesting to see whether reproducibility varies between different regions in the thoracic aorta.

We point out that the results of our analysis refer to measurements performed by beginners. Experienced readers might have obtained a lower variability of results. However, we have tried to exclude bias due to familiarity with one or another software to create fair comparison conditions for all programs.

We modified the equivalence test proposed by Zange et al. [15] to assess equivalence of different software solutions. Neither of the tests are validated, and different thresholds could significantly change the results.

The scans on both 3 T CMR scanners were performed on different days. Hence, the impact of physiological changes cannot be differentiated from the measurement error between both scanners. However, a dedicated analysis of the measurement error between scanners was not the main goal of this study. This comparison was only undertaken to allow interpretation of software comparison results in a larger context. A dedicated study focusing on scanner comparison should focus on consecutive scans in random order.

A final limitation of our study is the lack of a reference standard. After all, the aim of this study was to analyze comparability of software, not to evaluate accuracy of software using a reference standard.

## Conclusion

This study confirms that only SW-A and SW-C can be used equivalently for determination of SV, peak flow, and area. SW-D yielded also equivalent values for peak flow and area in comparison to SW-A and SW-C. Other software packages and parameters did not yield comparable results. Moreover, results from different scanners often showed better agreement than results from different software packages. High intra- and interreader variability for all parameters, especially for peak velocities, needs to be addressed. Before introducing 4D Flow CMR into clinical routine, not only scanning protocols but also postprocessing software need to be synchronized to allow for cross-vendor comparison at least of clinically relevant results.

## Data Availability

The datasets used and/or analyzed during the current study are available from the corresponding author on reasonable request. Readers are welcome to contact the corresponding author if they are interested in expanding this analysis with other software solutions or readers from other sites.

## Abbreviations

4D Flow CMR: Time-resolved 3-dimensional cardiovascular magnetic resonance
bwSV: Backward stroke value = Reflux
CMR: Cardiovascular magnetic resonance
MRI1: Ingenia, Philips Healthcare, Best, the Netherlands
MRI2: MAGNETOM Skyra, Siemens Healthineers, Erlangen, Germany
fwSV: Forward stroke volume
LLOA: Lower limit of agreement
LOA: 95% Limit of agreement
netSV: Net stroke volume
PC: Phase contrast
SW-A: Caas (v5.01, Pie Medical, Maastricht, Netherlands)
SW-B: Cvi42 (v5.9.4, Circle Cardiovascular Imaging, Alberta, Canada, Canada)
SW-C: GTFlow (v3.1.13, GyroTools, Zurich, Switzerland)
SW-D: MEVISFlow (v10.3, MEVIS Fraunhofer, Germany)
ULOA: Upper limit of agreement
V_avg_: Peak velocity averaged over a contour
V_max_: Peak maximum velocity of a contour, i.e. the highest velocity that was measured at one voxel in a contour during the cardiac cycle
WSS: Wall shear stress
WSS_con_: Peak wall shear stress value of wall shear stress averaged over a contour (“peak WSS per contour”)
WSS_seg_: Peak value of wall shear stress per segment, i.e., the highest wall shear stress that was measured in one of eight segments per contour (“peak WSS per segment”)
